# Myokine Circulating Levels in Postmenopausal Women with Overweight or Obesity: Effects of Resistance Training and/or DHA-Rich *n*-3 PUFA Supplementation

**DOI:** 10.3390/nu17152553

**Published:** 2025-08-05

**Authors:** Alejandro Martínez-Gayo, Elisa Félix-Soriano, Javier Ibáñez-Santos, Marisol García-Unciti, Pedro González-Muniesa, María J. Moreno-Aliaga, on behalf of OBELEX Project

**Affiliations:** 1 Department of Nutrition, Food Sciences and Physiology, Centre for Nutrition Research, Faculty of Pharmacy and Nutrition, University of Navarra, 31008 Pamplona, Spain; amartinez.104@alumni.unav.es (A.M.-G.); elisafelix93@gmail.com (E.F.-S.); mgarcia@unav.es (M.G.-U.); 2 Studies, Research and Sports Medicine Centre (CEIMD), Government of Navarre, 31005 Pamplona, Spain; javieribanezsantos0@gmail.com; 3 IdISNA, Navarra Institute for Health Research, 31008 Pamplona, Spain; 4 CIBER Physiopathology of Obesity and Nutrition (CIBEROBN), Carlos III Health Institute (ISCIII), 28029 Madrid, Spain

**Keywords:** myokines, DHA, muscle, resistance training, omega-3, postmenopausal, women, overweight, obesity

## Abstract

Background: Menopause increases the risk of cardiovascular diseases (CVD) accompanied by a decline in muscle function. Myokines, released by skeletal muscle, could play a significant role in cardiovascular health. Objectives and Methods: This study aimed to investigate the changes induced by a 16-week resistance training (RT) program and/or the docosahexaenoic acid (DHA)-rich *n*-3 PUFA supplementation on myokine and cytokine circulating levels and to study their associations with parameters of body composition, muscle function, and glucose and lipid serum markers in postmenopausal women with overweight/obesity. Results: At baseline, interleukin-6 (IL-6) levels were positively correlated with body fat and with tumor necrosis factor-alpha (TNF-α) levels and negatively associated with meterorin-like (METRNL) levels. Moreover, METRNL was inversely associated with insulin levels and with HOMA-IR. After the intervention, muscle quality improved with either treatment but more notably in response to RT. *N*-3 supplementation caused significant improvements in cardiometabolic health markers. TNF-α decreased in all experimental groups. Myostatin levels decreased in the RT and in the *n*-3 groups, and IL-6 increased in the *n*-3+RT group. Lastly, no interactions between treatments were observed. Conclusions: In postmenopausal women with overweight or obesity, RT could help improve skeletal muscle function, while DHA-rich *n*-3 supplementation might decrease CVD risk and might potentially improve muscle function. The modulation of myokine levels could be underlying some of the effects of DHA or RT; however, further research is necessary.

## 1. Introduction

Obesity is considered a worldwide pandemic [1], while life expectancy has increased significantly over the last century [2], with the number of people over 80 years old being expected to triple from 2019 to 2050 [3]. Aging is associated with body fat accumulation and with loss of skeletal muscle mass [4,5], and both aging and obesity seem to induce skeletal muscle dysfunction [5,6].

In women, the postmenopausal period is characterized by profound changes in metabolic health and body composition, such as increased chronic inflammation [7], increments in the accumulation of visceral adipose tissue [8], and losses of skeletal muscle mass and function [9], and it is a particular context in which aging and obesity tend to concur [10,11]. All things considered, the prevention of muscle loss and function during menopause would help reduce the risk of frailty, metabolic health-related diseases, and all-cause mortality, as well as improve independence and quality of life [12]. Thus, the search for strategies for the maintenance of adequate skeletal muscle functioning in the postmenopausal period seems crucial.

Skeletal muscle is responsible for many important bodily functions such as force production and breathing, and it is involved in nutrient storage, metabolism, and heat production, among many other processes [13]. Skeletal muscle is also considered the main site of insulin-mediated glucose uptake [14]. Moreover, it is now well established that skeletal muscle is a secretory organ. Upon muscle contractions, several molecules called exerkines are released from muscle and other organs [15,16]. Those molecules produced and released by skeletal muscle are called myokines [16,17]. Myokines such as irisin and meteorin-like (METRNL) have been proposed as the main drivers of the positive effects exerted by physical exercise [15,16,18].

Physical exercise has been shown to produce remarkable effects on skeletal muscle metabolism, mass, and function [19], to improve overall health, and to reduce the risk of several diseases [20]. Previous studies of our group and others have suggested that resistance training (RT) could be an effective strategy to counteract the menopause- and age-related loss of muscle mass, quality, and function, in parallel with improvements in cardiometabolic health markers [21,22]. Moreover, some studies have shown that omega-3 polyunsaturated fatty acids (*n*-3 PUFAs) administration could help to prevent muscle function and cardiometabolic health during aging and in postmenopausal women [22,23,24].

However, few studies have analyzed the relationship between circulating myokines, muscle mass and function, and cardiometabolic health in the postmenopausal status [25]. It has been suggested that regulating the levels of some of these myokines could be a therapeutic strategy for preventing sarcopenia, osteoporosis, and metabolic syndrome risk in postmenopausal women [26,27].

In this context, the first aim of this study was to investigate the associations between the circulating levels of several myokines and cytokines (Tumor necrosis factor–alpha (TNF-α), Interleukin-6 (IL-6), METRNL, myostatin, and irisin) and several parameters of body composition, muscle function, and cardiometabolic health biomarkers in postmenopausal women with overweight/obesity. Moreover, we aimed to study the changes induced by a 16-week progressive RT program and/or the regular supplementation with *n*-3 PUFAs, rich in docosahexaenoic acid (DHA).

## 2. Materials and Methods

### 2.1. Participants

Postmenopausal women aged between 55 and 70 years and with BMIs between 27.5 and 35 kg/m^2^ were recruited by phone calls using the database of the Nutritional Intervention Unit (NIU) of the Center for Nutrition Research of the University of Navarra (CIN-UNAV) and by advertisement in local newspapers. The inclusion criteria were age from 55 to 70 years old, body mass index (BMI) of 27.5–35 kg/m^2^ (overweight/type 1 obesity), stable weight within 3 previous months (±3 kg), and an adequate physical and physiological state to engage in resistance training. The exclusion criteria were use of regular prescription medication (including statins, antidiabetic drugs, proton pump inhibitors, and hormone replacement therapy. Additionally, antihypertensive, thyroid, anxiolytic, and antidepressant medications were considered as exclusion criteria if the dosage had been modified in the 3 months prior), any severe metabolic, hepatic, renal, cardiovascular, neuromuscular, arthritic, pulmonary, or other debilitating diseases, following special diets 3 months prior to the start of the trial, having suffered from eating disorders, surgically-treated obesity, or a history of alcohol or drug abuse.

Volunteers were assessed for eligibility. Before inclusion in the study, all candidates were screened using an extensive medical history that included blood biochemical data, resting electrocardiogram, and blood pressure measurements, conducted at the NIU of the CIN-UNAV. Participants were informed in detail about the possible risks and benefits of the study and gave their written informed consent prior to being enrolled in the study. The study was approved by the Research Ethics Committee of the University of Navarra (140/2015mod1 and 2015.140mod2) and conducted in compliance with the Helsinki Declaration guidelines.

### 2.2. Intervention Design

The intervention was a randomized double-blind placebo-controlled trial (RCT), registered at clinicaltrials.gov as NCT03300388.

Participants were allocated into four experimental groups for 16 weeks: (1) the placebo group (P) received placebo capsules containing olive oil (6 capsules of 0.5 g), (2) the omega-3 group (*n*-3) received DHA-rich fish oil concentrate capsules providing 1650 mg/day of DHA and 150 mg/day of EPA as ethyl esters, with a total content of 1950 mg/day of omega-3 PUFA, distributed in 6 capsules of 0.5 g of fish oil concentrate each, (3) the placebo + resistance training group (P+RT) received 6 placebo capsules and followed a progressive RT program of 2 sessions per week, and (4) the omega-3 + resistance training group (*n*-3+RT) received the 6 DHA-rich fish oil capsules containing 1650 mg/day of DHA and 150 mg/day of EPA, and followed a progressive RT program of 2 sessions per week.

Once the screening was completed, volunteers were randomly allocated to one of the four groups using the online software platform MATLAB^®^ (The MathWorks™, Natick, MA, USA). Randomization criteria were age and BMI according to the World Health Organization classification. The volunteers were randomized to create similar groups depending on whether they belonged to a group of age classified as adult or older adult (55–59 and 60–70 years old, respectively) [28]; and to a BMI of overweight Grade II or obesity Type I (27.5–29.9 and 30–35 kg/m^2^, respectively) [29].

At baseline and at the end of the trial, participants attended the NIU at the CIN-UNAV under 8–12 h fasting conditions, where anthropometric measurements, body composition data, and blood pressure determinations were carried out by a dietitian and a nurse. Basal fasting blood samples were then extracted in order to obtain serum/plasma to measure biochemical parameters.

At the end of the baseline visit, volunteers were given written dietary recommendations based on the guidelines from the Spanish Society of Community Nutrition (SENC) [30]. Follow-up dietary consultations were scheduled every two weeks. Dietary patterns were evaluated at baseline and at the end of the study with a validated 14-item questionnaire in order to assess adherence to the Mediterranean diet [31] and with a food frequency questionnaire (FFQ) [32] in order to evaluate potential changes during the intervention. When the baseline visit was completed, volunteers were given the corresponding supplements. Subjects were asked to report any side effects in order to evaluate any possible negative reactions associated with the consumption of the capsules. Once the baseline visit of the trial was completed and in every follow-up visit, all intervention groups received two boxes containing 6 blisters with 10 capsules each, for a total of 120 capsules. Participants were asked to return boxes in every follow-up visit to evaluate adherence to supplementation by leftover pill count.

Participants consumed two capsules with each meal (breakfast, lunch, and dinner). The placebo and the DHA-rich fish oil concentrate capsules (DHA^SCC^ premium) were provided by Solutex^®^ (Madrid, Spain). The DHA capsules contained tocopherol extracts as antioxidants to protect the highly unsaturated fatty acids from oxidation and also small amounts of silicon dioxide as a stabilizer. In order to standardize both types of capsules, the same quantity of tocopherols was added to the olive oil capsules. The low amount of the other stabilizer was not expected to have any significant effects or modify the health benefits of the fish oil concentrate. In order to ensure that the DHA supplements were not oxidized, peroxide and anisidine values were tested during the study and were considered adequate.

The dose of DHA-rich fish oil-derived supplement was selected based in previous studies [33,34,35] and in accordance with the United States Food and Drug Administration (FDA) recommendations of not exceeding 3 g/day of EPA and DHA, with up to 2 g/day coming from dietary supplements [36]. In order to fulfill these criteria, fish consumption was controlled, depending on their *n*-3 PUFA composition according to the European Food Safety Authority (EFSA) recommendations for normal cardiac function (250 mg/day), on the food composition tables from Mataix-Verdú et al. [37], and based on online food composition databases (Easy Diet^®^ and Odimet^®^ software, Spain). The consumption of *n*-3 PUFA-enriched food and dietary supplements was not allowed during the study [22].

Physical activity (PA) was controlled with a validated PA questionnaire [38] filled by participants at baseline and during the final visit. In order to obtain a direct comparison of PA between the four study groups, participants were asked to wear an accelerometer (ActiGraph GT3X, Actigraph Corporation, Pensacola, FL, USA) during a complete and randomly selected week of the study. The accelerometer was programmed for the subject’s gender, age, weight, height, race, and for the position it was worn on the body. The participants were instructed not to change their physical activity habits during the trial [22].

### 2.3. Resistance Training Program

Subjects allocated in the RT groups were asked to attend the Studies, Research, and Sports Medicine Centre training facilities (CEIMD), twice a week during the 16 weeks of intervention, to perform dynamic resistance exercise [39,40]. Eight exercises for the upper and lower main muscular groups were included in the training program. Two routines were designed with six exercises each. Four exercises (leg press, chest press, knee extension, and lat pull-down) were maintained along the RT program, while shoulder press and hip extension (Routine 1) and chest fly and leg curl (Routine 2) were selected to complete each routine, alternating between one routine and the other every two weeks. All exercises were conducted with the use of machines instead of free weights. With the exemption of hip extensions, all exercises were bilateral (using both limbs at the same time). Before testing and training, subjects attended three sessions in order to learn adequate technique and to become familiarized with the machines and with voluntary force production.

Strength tests were performed at the beginning, at the middle, and at the end of the trial, to obtain strength gains/losses data and to adjust training loads to the strength of each volunteer. The 1-repetition maximum (1-RM) approach was used. After a warm-up, a participant was asked to perform 1 repetition with a certain weight. When a repetition was completed, the participant was asked to perform another repetition after a rest period, with the weights being progressively increased in each repetition based on previous performance and perceived effort. This process was repeated until the participant could no longer complete the repetition with an adequate technique or could not execute it at all. The maximum weight lifted successfully in each exercise was considered the 1-RM.

The training progression was established using the pyramidal training approach. An intensity of 50% was selected as a starting point to the training program, and a maximum intensity of 80% was reached at week 10 [41]. Three to four series per exercise were performed in each training session, with 8–15 repetitions depending on the training loads. At least one of the researchers was present in every training session, in order to direct and assist each subject towards ensuring adequate performance in each exercise following American College of Sports Medicine (ACSM) guidelines for older adults.

To control for strength gains or losses in the untrained groups, first and last follow-up visits were scheduled at the training facilities for subjects allocated to these groups in order to perform 1-RM tests (including their corresponding familiarization sessions). Muscle quality was used as an indicator of relative strength. As previously reported by Ribeiro et al. [42], muscle quality was calculated as the sum of the 1-RM lifted in the chest press and leg press exercises (kg) divided by total skeletal muscle mass (kg).

### 2.4. Evaluation of Body Composition

Body composition was analyzed at baseline and at the end of trial by total and segmented dual-energy X-ray absorptiometry (Lunar iDXA, encore 14.5, Madison, WI, USA), as reported previously [43]. Body fat and lean body mass (total and as a percentage of body weight) were measured. Skeletal muscle mass (SMM) was estimated using the prediction equation developed by Kim et al. [44], and was calculated as follows:

SMM = (1.18 × ALST [kg]) − (0.03 × age [years]) − 0.14, LST = arms lean mass “[kg]” + legs lean mass “[kg]”, ALST: Appendicular lean soft tissue.

### 2.5. Evaluation of Serum Biomarkers

Blood samples were extracted at baseline and after the intervention, they were centrifuged at 1500× *g* for 15 min at 4 °C, and aliquots of serum/plasma were frozen at −80 °C until analysis. Fasting serum lipid and glucose metabolism biomarkers, including total cholesterol, HDL-cholesterol, triglycerides (TGs), and glucose levels were determined at baseline and at the end of the intervention on an autoanalyzer (Pentra C-200; HORIBA ABX, Madrid, Spain) following the manufacturer’s instructions. LDL-cholesterol and VLDL-cholesterol were calculated using Friedewald’s equations [45]. The atherogenic index was calculated as Log (TG/HDL), as described by Fernández-Macías et al. [46]. Fasting insulin levels were determined with an ELISA kit (#10-1132-01, Mercodia, Uppsala, Sweden) on an autoanalyzer (Triturus ELISA Instrument, Grifols, Barcelona, Spain) following the manufacturer’s instructions. The homeostasis model assessment of insulin resistance (HOMA-IR) index and the homeostasis model assessment of beta cell function (HOMA-β) index were calculated as described previously [47,48].

Serum myokine and cytokine levels were analyzed at baseline and at the end of the intervention using enzyme-linked immunosorbent assay (ELISA) kits, following the instructions of their respective manufacturers. IL-6, METRNL, and irisin were analyzed using Human IL-6 DuoSet^®^ ELISA #DY206-05, Human Meteorin-like/METRNL DuoSet^®^ ELISA #DY7867-05, and Human Irisin/FNDC5 DuoSet^®^ ELISA #DY9420-05, respectively (R&D Systems, Minneapolis, MN, USA). Myostatin was analyzed with the Human GDF-8/Myostatin ELISA Kit (#EH215RBX5, Invitrogen, Thermo Fisher Scientific, Waltham, MA, USA), and TNF-α was analyzed on an autoanalyzer (Triturus ELISA Instrument, Grifols, Barcelona, Spain), using Human TNF-alpha Quantikine HS ELISA (R&D Systems, Minneapolis, MN, USA).

### 2.6. Statistical Analysis

Losses in fat mass were considered the primary outcome. As reported by Félix-Soriano et al. [22], the estimated effect size was 1.185, based on previous studies on the combination of *n*-3 supplementation and exercise [49,50]. Considering a bilateral alpha of 95% and a power calculation of 90%, the number of volunteers per group was set to 16. Based on previous reports [51,52,53], the expected dropout rate was 25%, and the final number of volunteers estimated was set at 20 per group.

Statistical analyses were performed using GraphPad Prism version 8.00 for Windows (GraphPad Software, La Jolla, CA, USA) and STATA 17 (Stata, College Station, TX, USA). The results were expressed as the mean (SD). The percentual changes in body fat and the changes in skeletal muscle mass were identified as possible confounding variables and were used for adjustment in some parameters. Adjusted values were expressed as the mean ± SEM.

The normality of residuals was studied with the Shapiro–Wilk test in order to select the appropriate statistical test. The comparisons between groups at baseline were conducted by either one-way ANOVA or the Kruskal–Wallis test. The comparison between values at baseline and at the end of the intervention in each group were assessed by either the paired *t*-test or Wilcoxon signed-rank test, depending on normality.

Due to the factorial design of the study, two-way ANOVA was used in order to analyze the changes produced by DHA-rich *n*-3 PUFA supplementation (*n*-3) and resistance training (RT), as well as the possible interactions between both treatments (*n*-3xRT). When a statistically significant interaction was detected, contrast analyses were performed in order to differentiate the group effects. When the interaction did not reach statistical significance, the main effects were studied. The analysis of main effects allowed us to differentiate the changes observed in the groups receiving one treatment (*n*-3 or RT) from the groups not receiving such treatment, whether they were allocated to the other treatment group or not (*n*-3: *n*-3-supplemented vs. P-supplemented groups; RT: RT vs. non-RT groups).

Correlations between myokine levels and the other parameters were conducted using either Pearson correlation coefficient test or Spearman’s rank coefficient test, depending on normality. After the correlation tests were completed, the Benjamini–Hochberg procedure was applied in order to detect false positive correlations. The false discovery rate (FDR) was set at 5% (or 0.05), and the *p* values reported in the correlations corresponded to the q values (or adjusted *p* values) obtained with this procedure. Statistical significance for all tests was set at two-tailed *p* < 0.05.

## 3. Results

### 3.1. Basal Characteristics

A total of 124 postmenopausal women were screened. Of those, 85 were included in the study, and 71 of them finished the intervention [22]. A flow chart of participants can be found in Figure S1. The baseline anthropometric and biochemical data from all 71 participants that completed the study are presented in Table 1 and Table 2, respectively. As Table 1 shows, the participants were 58.5 ± 3.2 years old, with a body weight of 78.87 ± 7.35 kg. They had a mean BMI of 30.61 ± 2.12 kg/m^2^, and their percentage of body fat mass was 46.76 ± 3.29%, with both parameters classifying the whole population as obese.

As presented in Table 2, fasting glucose levels were in the prediabetic range, slightly over the normal range (104.90 ± 15.50 mg/dL). Total and LDL-cholesterol were in the borderline high ranges (245.60 ± 37.50 and 160.36 ± 34.42 mg/dL, respectively), while their mean HDL-cholesterol and TGs (64.28 ± 14.70 and 105.10 ± 43.60 mg/dL, respectively) were in the desirable/normal ranges. The circulating levels from all measured myokines/cytokines were within previously reported ranges [54,55,56,57,58].

### 3.2. Correlations Between Myokine/Cytokine Levels and Basal Characteristics of the Subjects

**Figure 1:**
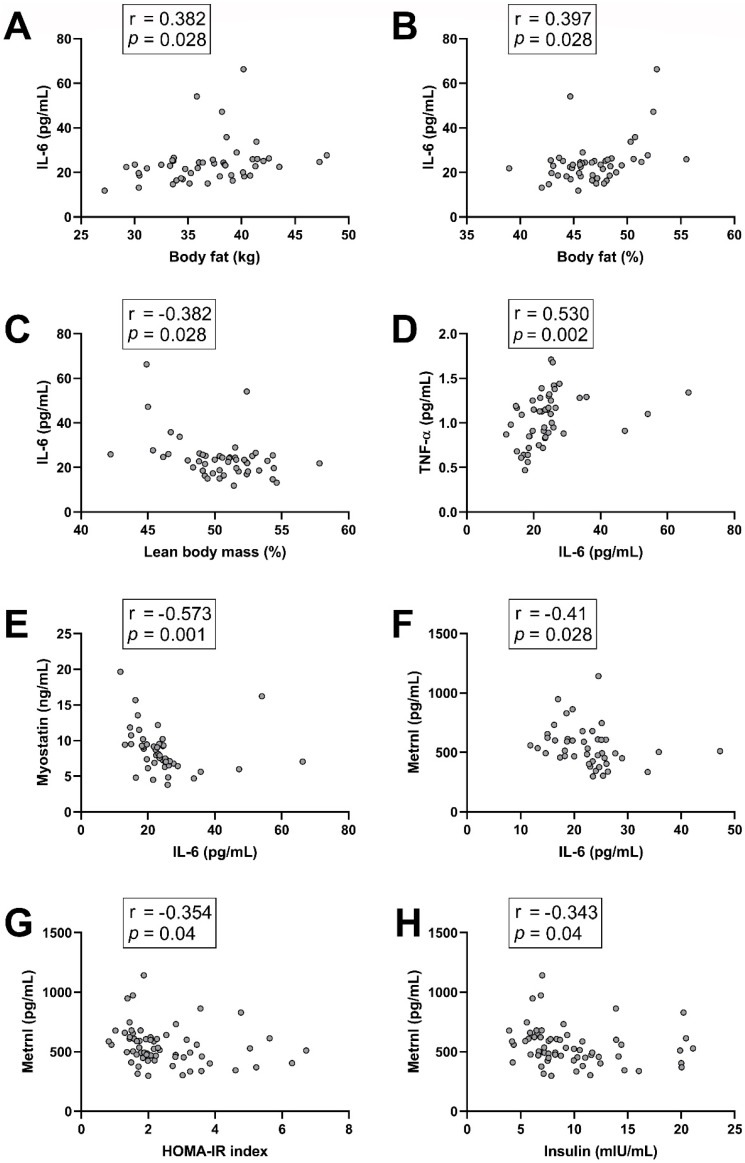
Correlations between circulating myokine/cytokine levels and participant’s baseline characteristics. (**A**) IL-6 vs. Body fat (kg); (**B**) IL-6 vs. Body fat (%); (**C**) IL-6 vs. Lean Body mass (%); (**D**) TNF-α vs. IL-6; (**E**) Myostatin vs. IL-6; (**F**) Metrnl vs. IL-6; (**G**) Metrnl vs. HOMA-IR index; (**H**) Metrnl vs. Insulin. Spearman’s rank correlation coefficient (r) and *p* values are shown above their corresponding figures. IL-6: Interleukin-6; TNF-α: Tumor necrosis factor-alpha; METRNL: meteorin-like; HOMA-IR: homeostasis model assessment of insulin resistance.

First, we evaluated the possible associations between circulating myokine levels at baseline in all subjects, as well as the possible associations between circulating myokine levels and the other population characteristics reported. All the results that were revealed to be statistically significant by the correlation analysis after conducting the Benjamini–Hochberg correction are presented in Figure 1.

The pleiotropic cytokine/myokine IL-6 is habitually considered to have pro-inflammatory properties [59]. IL-6 was correlated with three variables of body composition, as shown in Figure 1A–C. It was positively correlated with total body fat (kg) and with the percentage of body fat (r = 0.382, *p* = 0.028 and r = 0.397, *p* = 0.028, respectively), and it was negatively correlated with the percentage of lean body mass (r = −0.382, *p* = 0.028). In addition, the levels of three other cytokines/myokines were significantly correlated with IL-6. As shown in Figure 1D, the circulating levels of the pro-inflammatory cytokine TNF-α demonstrated a positive correlation with IL-6 levels (r = 0.530, *p* = 0.002). On the other hand, the levels of myostatin and the levels of the anti-inflammatory METRNL were negatively correlated with IL-6 circulating levels (r = −0.573, *p* = 0.001 and r = −0.41, *p* = 0.028, respectively) (Figure 1E,F). Interestingly, METRNL, which has been proposed to have insulin sensitizing properties [60,61], was correlated with two variables related to glucose homeostasis. It was negatively correlated with the index of insulin resistance (HOMA-IR), and also with basal insulin levels (r = −0.354, *p* = 0.04 and r = −0.343, *p* = 0.04, respectively) (Figure 1G,H). No significant correlations were found between irisin levels and the other variables studied.

### 3.3. Baseline Anthropometric and Biochemical Characteristics of the Different Experimental Groups

The data from the participants at baseline, allocated into the four experimental groups, are presented in Supplementary Table S1. No statistically significant differences were observed between groups in any variable, with the sole exception of METRNL (*p* = 0.003). Its circulating levels were significantly lower at baseline in the *n*-3+RT group compared with the P and the P+RT groups.

### 3.4. Effects of the 16-Week Intervention with DHA-Rich n-3 PUFA Supplementation and/or Resistance Training in Postmenopausal Women with Overweight/Obesity

#### 3.4.1. Effects on Myokines/Cytokines

Subsequently, we examined the changes produced by *n*-3 supplementation and/or RT on the circulating levels of the different cytokines/myokines. The possible interactions between treatments (*n*-3 supplementation and RT), as well as the analysis of the main effects of the treatments, were studied using two-way ANOVA.

**Figure 2:**
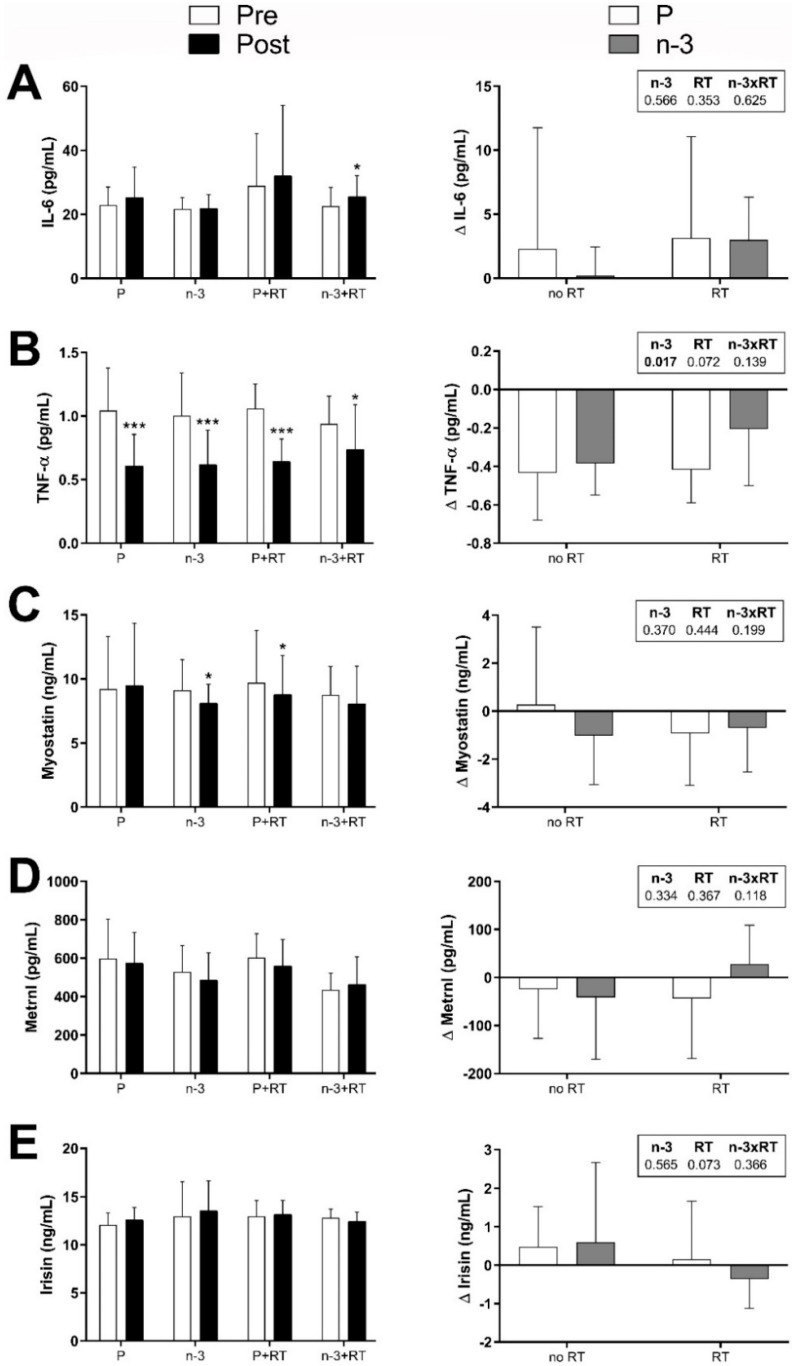
Effects of 16 weeks of DHA-rich *n*-3 PUFA supplementation and/or resistance training on myokine/cytokine circulating levels in postmenopausal women with overweight/obesity. Effects on: (**A**) IL-6; (**B**) TNF-α; (**C**) Myostatin; (**D**) Metrnl and (**E**) Irisin. P: placebo group; *n*-3: DHA-rich *n*-3 PUFA supplemented group; P+RT: placebo + resistance training group; *n*-3+RT: DHA-rich *n*-3 PUFA supplemented + resistance training group; TNF-α: Tumor necrosis factor-alpha; IL-6: Interleukin-6; METRNL: meteorin-like. *N* per group: P = 20; *n*-3 = 15; P+RT = 20; *n*-3+RT = 16; number of undetermined values: IL-6 = 22; METRNL = 6; myostatin = 1; irisin = 8; *n* per group: P = 17–20; *n*-3 = 10–15; P+RT = 13–20; *n*-3+RT = 12–16. Data are presented as mean (SD). Left panels: comparison between myokine levels before (Pre) and after the intervention (Post) analyzed by Paired Student’s *t*-test or Wilcoxon’s signed-rank test. Right panels: changes after the intervention (Post–Pre) between groups analyzed by two-way ANOVA. The *p* values for the main factors of study, *n*-3 supplementation (*n*-3), exercise (RT), and the interaction between both (*n*-3xRT) appears under the corresponding column, with significant *p* values in bold. Statistical significance indicates a main effect for such factor (*n*-3: *n*-3-supplemented vs. P-supplemented groups; RT: RT vs. non-RT groups). * *p* < 0.05, *** *p* < 0.001 vs. baseline.

The changes observed in all the measured myokines/cytokines after the 16-week intervention are presented in Figure 2. The left panels show the comparison between their circulating levels before (Pre) and after the intervention (Post), for each experimental group. The right panels show the changes in circulating levels at the end of the intervention (Post–Pre).

A significant increase in IL-6 circulating levels was observed after the intervention in the group that combined RT and *n*-3 supplementation (*p* = 0.010), while no significant changes were observed within the other groups (Figure 2A). However, no significant effects for *n*-3 or for RT were detected in the analysis between groups.

TNF-α levels decreased significantly in all groups at the end of the intervention, including the placebo group (*n*-3+RT: *p* = 0.035; other groups: *p* < 0.001), as shown in Figure 2B, left panel. Curiously, the two-way ANOVA revealed a main effect of *n*-3, with both *n*-3-supplemented groups (with or without RT) showing a less pronounced decrease in the levels of TNF-α (*p* = 0.017 vs. P-supplemented groups).

Serum myostatin levels were significantly reduced in the *n*-3-supplemented group and in the RT group after the intervention (*p* = 0.018 and *p* = 0.036, respectively) (Figure 2C, left panel), while no changes were observed in the other groups, including the group combining both treatments. However, the analysis between groups revealed no significant differences in the changes of myostatin levels between groups (Figure 2C, right panel).

Concerning the circulating levels of METRNL and irisin, no statistically significant changes were observed after the intervention either within groups or between groups (Figure 2D,E).

When the changes in the circulating levels of all myokines (IL-6, METRNL, myostatin, and irisin) were adjusted for the changes in skeletal muscle mass, no notable differences were observed in comparison with the unadjusted analysis, with one exception. In the adjusted analysis of irisin levels, a tendency towards a statistically significant main effect of RT was found in the analysis between groups via ANOVA (*p* = 0.051 vs. non-RT groups).

#### 3.4.2. Effects on Muscle Function and Serum Biochemical Parameters

The effects of the 16-week intervention on the variables related to body composition, muscle function, and the markers of glycemic control and serum lipid metabolism are presented in Table 3.

Regarding body composition, the total body fat decreased significantly in all groups, including the placebo. However, no differences between groups were detected. As reported by Félix-Soriano et al. [22], all four groups also showed reductions in BMI, BW, and the percentage of fat mass. They also reported increases in the percentage of lean mass. However, in the present study, no significant changes were observed in any group after the intervention neither in total lean body mass nor in skeletal muscle mass, and no significant differences between groups were detected.

In terms of muscle function, muscle quality was used as a surrogate for relative strength. Except for the placebo group, all the other groups (*n*-3 and/or RT) showed significant increments in muscle quality after the 16 weeks. The analysis between groups revealed a significant main effect of RT. Thus, both RT groups showed a significant increase in muscle quality after the intervention (*p* < 0.001 vs. non-RT groups). Interestingly, a main effect was also detected in *n*-3, with significant increases in muscle quality in the *n*-3-supplemented groups (*p* = 0.011 vs. P-supplemented groups).

As for glucose homeostasis, our group has previously reported [22] a tendency (*p* = 0.066) towards a decrease in the glucose area under the curve (AUC) caused by RT and a significant decrease in the case of the incremental AUC (iAUC). Here, we included the homeostasis model assessment of β-cell function (HOMA-β index) as a marker of pancreatic β-cell function and insulin resistance, but no statistically significant changes were observed in this parameter.

Regarding markers of serum lipid profile and cardiometabolic risk, we included very-low-density lipoprotein (VLDL)-cholesterol and the atherogenic index. All groups except the placebo showed significant decreases in VLDL-cholesterol levels after the intervention. A significant main effect was detected by two-way ANOVA analysis, showing more notable decreases in VLDL-cholesterol levels in the groups receiving *n*-3 PUFA when compared with P-supplemented groups (*p* = 0.047). With respect to the atherogenic index, only the *n*-3 group showed a significant decrease after the intervention. Similarly to VLDL-cholesterol, the groups supplemented with *n*-3 had more marked decreases in the atherogenic index than the P-supplemented groups (*p* = 0.040).

When serum lipid markers were adjusted for the relative losses in body fat, the main effects of *n*-3 supplementation on VLDL-c levels and on the atherogenic index, as well as the comparisons between the levels of these variables before and after the intervention, remained statistically significant. In addition, a tendency towards statistical significance was observed in the interaction between treatments in the case of VLDL-c (*p* = 0.051).

Noteworthy, no significant interactions between *n*-3 supplementation and RT were detected in any of the analyzed parameters.

#### 3.4.3. Correlations Between Changes in Myokine Levels and Changes in Biochemical Parameters After the Intervention

Lastly, in order to investigate further the potential associations between the changes in the circulating levels of myokines/cytokines after the intervention with changes in the participant’s metabolic characteristics, a correlation analysis was performed in each of the four experimental groups.

**Figure 3:**
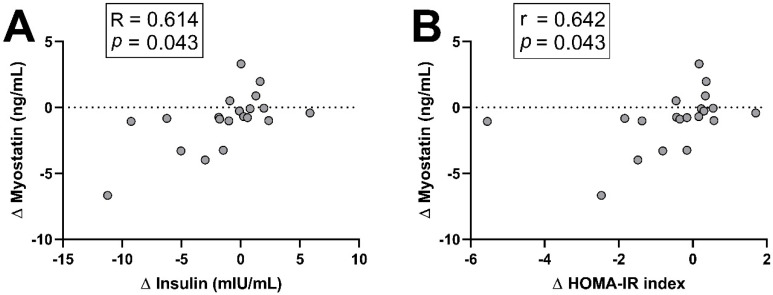
Correlations between changes observed in myostatin circulating levels and changes in fasting insulin levels (**A**), as well as changes in HOMA-IR (**B**) after 16 weeks of DHA-rich *n*-3 PUFA supplementation and/or resistance training (RT) in postmenopausal women with overweight/obesity. Both correlations correspond to changes in the RT group. R: Pearson correlation coefficient, r: Spearman’s rank correlation coefficient. HOMA-IR: homeostasis model assessment of insulin resistance.

Only two statistically significant results were obtained, both in the RT-alone group. As shown in Figure 3A,B, the changes in myostatin levels after the intervention were positively correlated with the changes in insulin levels and also positively correlated with the changes in the HOMA-IR index, a marker of insulin resistance.

## 4. Discussion

This study focused on investigating the effects of a 16-week intervention with DHA-rich omega-3 PUFA supplementation and/or a progressive RT program on the circulating levels of four myokines (IL-6, METRNL, myostatin, and irisin), the cytokine TNF-α, and on certain parameters of body composition, muscle function, and serum markers of glucose and lipid metabolism in postmenopausal women with overweight or obesity. Notably, a previous article of our group by Félix-Soriano et al. [22] reported changes in several parameters from the same intervention. Hence, some of our present findings will be discussed in the context of those previous results.

First, we studied the baseline circulating myokine/cytokine levels of the 71 participants that completed the intervention (grouping all of them into a single population), as well as the possible correlations between myokine/cytokine levels and the aforementioned parameters.

Acute or transient increases in IL-6 circulating levels have been described to produce positive effects [62], while chronic increases have detrimental and pro-inflammatory effects [63]. In fact, elevated IL-6 levels are considered a marker of chronic inflammation [64]. Interestingly, IL-6 levels at baseline were positively correlated with body fat (total and as a percentage of body weight). Our findings, as well as similar correlations reported in the literature [65], seem coherent with the well-established link between obesity and chronic inflammation [66]. Furthermore, IL-6 levels were negatively correlated with the percentage of lean body mass (LBM), a finding reinforced by a meta-analysis in which inverse correlations between IL-6 levels and muscle mass and muscle function were observed in several studies [67]. In addition, TNF-α levels were positively correlated with IL-6. Their positive correlation has been previously reported [68] and seems reasonable considering that TNF-α is considered the cytokine with the most notable implications in chronic inflammation [69], a condition of which both IL-6 and TNF-α are considered biomarkers [64]. Therefore, it seems plausible that both factors could have related circulating levels, especially in the context of increased adiposity and/or menopause, conditions that promote a pro-inflammatory state, in which the levels of both IL-6 and TNF-α are typically increased [7,66]. On the contrary, our data have shown that METRNL levels had a negative correlation with IL-6, which is in agreement with previous studies in other populations [70] and with the fact that METRNL is considered as an anti-inflammatory myokine [71]. Furthermore, METRNL has been negatively associated with adiposity, IR, and VAT [72,73], while IL-6 is usually elevated in all of them, and it was positively correlated with body fat in our study, reinforcing the likelihood of the negative correlation between METRNL and IL-6. Myostatin was also negatively correlated with IL-6 levels, a correlation already reported in the literature, which seems to be related to muscle mass [74]. Thus, in postmenopausal women with overweight/obesity, decreases in METRNL levels and increased IL-6 levels could be expected, which might explain the negative correlations observed between them. Taking everything into consideration, these findings suggest that body composition might play a crucial role in the regulation of cytokines/myokines related to chronic inflammation; thus, having a healthy body composition could be key in order to control this exceptionally deleterious condition.

In addition, we also observed that METRNL circulating levels were inversely correlated with fasting insulin levels and also with the HOMA-IR index, a correlation already reported in a recent meta-analysis [75]. Interestingly, METRNL has been proposed to have positive effects on glucose homeostasis [60,76], and METRNL levels have been positively associated with insulin sensitivity [61] and negatively correlated with HbA1c [77], and several studies have reported lower circulating levels in people with obesity, overweight, prediabetes, and T2DM, in comparison with controls [70,72,77,78,79,80]. Two meta-analysis [75,81] found no significant associations with T2DM, but they reported inverse correlations between METRNL and adiposity. Hence, our results might support a potential involvement of this myokine in glucose homeostasis and suggest that METRNL could be a potential predictive biomarker of insulin sensitivity and metabolic flexibility in postmenopausal women with overweight/obesity. This agrees with several studies [75,76]; however, the literature shows some controversial results, and the roles of METRNL need to be further investigated.

Then, we studied the effects of 16 weeks of DHA-rich *n*-3 PUFA supplementation and RT, alone or in combination, on the circulating myokine/cytokine levels and on parameters of body composition, muscle function, and biomarkers of serum lipid and glucose metabolism.

Concerning the parameters of body composition, all groups, including the placebo, showed reductions in total body fat after the intervention, although the dietary advice provided to all participants was not intended to induce losses in weight but to promote a healthy dietary pattern [22].

As for LBM, our group has previously reported that the percentage of LBM increased similarly in all groups after the intervention and also found a positive effect of RT in arms LBM, compared with the changes observed in the un-trained groups [22]. However, in the present study, no significant changes were found neither in total LBM measured by DEXA scan nor in the estimated skeletal muscle mass. Similar results have been described in postmenopausal women [82,83,84,85], but increases have also been reported [86,87,88]. Of note, the studies that reported increases either included both men and women, used less accurate methods of body composition assessment (electrical bioimpedance), had longer interventions, or did not observe decreases in weight among their participants, which might help explain the discrepancies. Moreover, the increase in muscle mass (hypertrophy) is based on muscle protein synthesis (MPS) being greater than muscle protein breakdown (MPB). It has been reported that MPS tends to decrease with age, obesity, and/or systemic inflammation, a phenomenon known as anabolic resistance [89], and also that the increases in MPB in response to RT are significantly higher in un-trained vs. trained individuals [90]. Because of that, it is conceivable that our participants might have experienced important increases in the rates of MPB as a result of RT, which coupled with a potentially decreased MPS response might have prevented hypertrophy from occurring. In young healthy un-trained individuals, it took 3 weeks of RT for the MPB to be sufficiently attenuated in order for hypertrophy to occur [91]. Additionally, protein is essential for muscle renewal and growth, and it has been shown to increase MPS, reduce MPB, and to work synergistically with RT towards hypertrophy [92]. High protein intakes (>1.4 g/kg/day) seem to be required in order to maximize muscle adaptations in response to RT [93,94], and studies focused on muscle changes in response to RT showed greater improvements in muscle mass or strength in the groups with higher protein intakes (around 1.5–2.5 times the RDA for this macronutrient) [93,95]. Moreover, protein requirements in order to stimulate MPS and adapt to RT increase with age [96] and during periods of caloric deficit [94,97,98]. In our study, considering the relevance of dietary protein intake in supporting muscle adaptation, protein intake was controlled using data from the FFQ at the beginning and at the end of the intervention. Data of the FFQ showed that protein intake was around 1.5–1.7 g/kg/day and that was similar between groups at the beginning and at the end of the intervention (Figure S2), suggesting that in our study dietary protein intake is not likely to be a confounding factor for RT effects. Although speculative, it seems plausible that the protein requirements in order to produce hypertrophy in our postmenopausal participants were remarkably high, and since no specific advice on protein intake was provided to the RT groups, we conjecture that the likelihood of those requirements having been met was not very strong.

Regarding *n*-3 PUFA supplementation, increases in MPS have been reported in older adults [99], and several lines of evidence suggest that their supplementation might increase MPS and enhance the anabolic response to RT [100]. A review of the topic [101] reported mixed results in the literature but pointed out that the studies that found no significant changes had durations under 12 weeks and doses below 2 g/day of combined EPA+DHA. Moreover, these studies used techniques that measured total LBM, such as DEXA or bioimpedance, methods that might not be precise enough to detect minor differences, albeit clinically significant ones, in skeletal muscle specifically. In order to avoid these possible limitations, a study was conducted using MRI and providing >3.3 g/day [24], and changes were detected in the volume of the thigh muscle at 6 months. Considering that RT, which is the main stimulus for muscle hypertrophy, caused no changes in total muscle mass in our intervention, and that some of the aforementioned limitations were present, we consider that the potential benefits of *n*-3 PUFAs on skeletal muscle mass should not be dismissed based on our findings.

Since losses of skeletal muscle mass are expected with age [5], strategies focused on promoting muscle hypertrophy seem crucial, however, muscle function might be even more relevant [12,102]. Muscle function is more strongly correlated with independence in older adults than muscle mass [103], and in fact, low muscle strength is now considered the main determinant of sarcopenia [104]. A previous article of our group reported significant increases in the muscle quality (relative strength) of upper and lower limbs in the RT groups [22]. In the present study, we summarized muscle quality into a single value, and we used predicted skeletal muscle mass [44] instead of LBM. We found that RT caused significant increases in muscle quality, similar to previous studies in postmenopausal women [83,105,106].

Interestingly, muscle quality was higher in the *n*-3-supplemented groups when compared with the P-supplemented ones. Several observational studies have reported positive correlations between *n*-3 PUFAs and muscle strength in older women [107,108,109], and an intervention in a similar population found significant improvements in muscle strength [24]. Lastly, *n*-3 PUFA supplementation in combination with RT has been shown to further improve muscle strength in elderly women compared with RT alone [110], but the interaction between treatments was not statistically significant in our study. Thus, we report that 16 weeks of RT and or supplementation with *n*-3 PUFA rich in DHA caused significant improvements in muscle function in postmenopausal women with overweight/obesity. Based on these findings, we suggest that both RT and the long-term supplementation with these FAs might be able to improve muscle function in this population.

Concerning lipid metabolism, all groups except the placebo showed reduced levels of VLDL-cholesterol, a marker associated with the risk of CVD [111,112]. This lipoprotein is the major carrier of serum TGs, independently associated with CVD [113], and our group has reported previously a significant decrease in this marker in the *n*-3-supplemented groups [22]. In addition, the atherogenic index, considered a reliable marker of CVD [114,115], improved significantly in the group supplemented with *n*-3 PUFA. Furthermore, the analysis between groups revealed a main effect of *n*-3 PUFA supplementation, with *n*-3-supplemented groups showing more notable improvements in both VLDL-c and in the atherogenic index than P-supplemented groups. Taking into account that an atherogenic lipid profile with increased VLDL-c and atherogenic index are hallmarks of the postmenopausal period [116,117], the improvements observed in those markers suggest that *n*-3 PUFA supplementation could potentially be a relevant strategy in order to decrease cardiovascular risk in this population. Coupled with previous studies, our results support that the regular practice of RT and/or *n*-3 PUFA supplementation could have important clinical implications in this population, by improving muscle quality, preventing sarcopenic obesity, and reducing cardiometabolic health risk markers, among others.

With regard to the changes in circulating cytokines/myokines, TNF-α levels decreased in all groups after the intervention, including the placebo group. A meta-analysis in older adults reported that decreases in TNF-α levels were found in all clinical trials in which body fat decreased, while no changes were observed in those trials in which body fat remained unchanged [88]. Moreover, both that meta-analysis and a previous one [118] reported that exercise interventions had no effects on TNF-α. Since TNF-α levels and body fat decreased in all groups, our findings seem to support that, indeed, fat loss could be the main driver of the decreases in this pro-inflammatory cytokine, independent from RT and *n*-3 supplementation. Unexpectedly, the reduction in TNF-α was lower in the *n*-3-supplemented groups when compared with the P-supplemented groups. Notably, olive oil was used as a placebo, and it has been associated with decreases in inflammatory biomarkers [119,120].

IL-6 levels increased in the *n*-3+RT group, while the analysis between groups found no significant effects of either treatment. Upon muscle contractions, skeletal muscle produces and liberates significant amounts of IL-6 into circulation. In fact, among all myokines, its increases are the first to be detected during exercise and the most pronounced [121]. Nevertheless, a consensus has not been reached yet in regard to exercise and chronic changes. A meta-analysis on exercise training found decreases in IL-6 levels [118], while another one analyzing RT interventions found a tendency (not significant) towards decreased levels [88]. This study suggested that the decreases in IL-6 might depend on the changes in muscle mass, which might explain why no changes were observed in the RT group. However, the increase observed in the *n*-3+RT group is hard to reconcile with the existing literature, especially when considering that *n*-3 PUFAs have been shown to have anti-inflammatory effects and to reduce IL-6 levels [23,122,123]. These increased levels of IL-6 are not likely to be a consequence of a transient response to exercise, since blood samples were collected 72 h after the last exercise bout, which should be enough time for IL-6 levels to return back to normal [124,125]. However, our current data seem to suggest that *n*-3 PUFA supplementation could affect the transiently increased levels of IL-6 induced by exercise. This transient increase in muscle-derived IL-6 induced by exercise has been proposed to be beneficial, as it has been shown to stimulate GLP-1 secretion, improving insulin secretion and glycemic control [126]. Indeed, we have previously shown that the RT program improved the response to the glucose tolerance test in subjects with and without *n*-3 PUFA supplementation. Therefore, these data highlight the relevance of carrying out future studies to better characterize this hypothesis and the potential interactions between acute and long-term interactions of *n*-3 PUFA supplementation and physical exercise, since both strategies can modulate similar potential metabolic pathways [127,128]; thus, overlapping and competing effects between treatments should not be ruled out and might have played a role in our results.

METRNL is induced in skeletal muscle in response to exercise [60]; however, in our intervention, no significant changes were detected in any group. A study reported increases in METRNL levels after 8 weeks of RT [129], but this study was conducted in T2DM patients. In fact, most studies on METRNL have been conducted either in T2DM patients or in people with varied degrees of adiposity and/or IR [77,78,130]. Therefore, METRNL levels might not fluctuate significantly in response to RT in the absence of marked impairments in glucose homeostasis.

Myostatin was the first discovered myokine and is considered a negative regulator of skeletal muscle growth [131,132]. We observed reduced myostatin levels after the intervention in the RT group and the *n*-3 group; however, no differences were detected for any treatment in the comparison between groups. The literature on myostatin levels shows decreases in men of different ages and metabolic conditions in response to RT [133,134,135], including a recent systematic review and meta-analysis that included men and women [136]. Regarding *n*-3 PUFAs, an 8-week intervention found no changes in myostatin levels in response to *n*-3 supplementation [137]. Interestingly, myostatin has been negatively correlated with muscle function in postmenopausal women, but not in men [138], which might partially explain the decreases in myostatin observed, considering that both treatments were able to improve muscle function. Thus, 16 weeks of *n*-3 supplementation and of RT alone, but not in combination, decreased myostatin circulating levels in postmenopausal women with overweight/obesity in the absence of changes in skeletal muscle mass. These findings seem to support the idea of myostatin levels not being necessarily tied to changes in lean mass [139] and seem to reinforce the negative association between myostatin levels and muscle function in postmenopausal women [138]. However, in our study we did not find any significant association between the changes in circulating myostatin and the changes in muscle quality, suggesting than other systemic metabolic factors could be the main drivers of the changes observed in myostatin. Indeed, in the RT group, the decreases in myostatin levels were correlated with the decreases in insulin levels and also with the decreases in the HOMA-IR index, which might suggest a possible implication of these factors in the regulation of myostatin production. However, a role of this myokine in mediating the effects of RT on glucose homeostasis cannot be ruled out, supporting in part the proposed involvement of this myokine in glucose control [139,140,141].

Regarding irisin, several studies have reported transient increases in its levels after exercise [142,143,144,145], while fewer have reported chronic increases [133,146]. Additionally, a remarkable number of studies involving diverse types of exercise and populations have reported no changes in circulating irisin levels [142,147,148,149,150,151]. Moreover, some of these authors [148,151] have suggested that irisin changes in response to exercise might be random and have questioned the notion of irisin being involved in the positive effects of exercise. We report that 16 weeks of RT did not cause any significant changes in irisin levels, an observation that seems to be in agreement with most studies, but our findings cannot confirm nor refute previous criticisms regarding irisin.

Overall, our results suggest that myokine changes might reflect changes in muscle function and/or changes in metabolic health, considering that changes in muscle mass did not differ between groups.

### Limitations and Future Perspectives

A longer trial could have been necessary in order to observe changes in muscle mass as well as more significant and long-term changes in myokine regulation. Moreover, individual characteristics, such as genetic polymorphisms, basal DHA levels, or overall fitness among others, would have had an influence on the response to the treatments, as reported in the literature [152,153,154,155]. In fact, a high degree of individual variability was found in myokine levels. Thus, further studies with larger sample size should be encouraged. Lastly, this study was solely focused on RT, and it is important to consider that other types of exercise might differ in regard to myokine regulation, especially in combination with *n*-3 PUFAs. In summary, longer trials, with larger sample sizes and including different types of exercise or combinations of them would be recommended in future trials. Furthermore, DHA and exercise have been proposed to share metabolic pathways, and thus, potential interactions and synergistic or antagonistic effects between treatments should be further characterized and taken into consideration in future research.

## 5. Conclusions

In summary, our data suggest relevant associations between myokines circulating levels with body composition and metabolic features in postmenopausal women with overweigh/obesity. Indeed, IL-6 basal levels were positively associated with adiposity and negatively associated with lean mass percentage. Our findings also suggest a positive role of METRNL in glucose homeostasis in these population. Furthermore, 16 weeks of resistance training were able to produce significant improvements in muscle quality in postmenopausal women with overweight or obesity. Thus, resistance training could help improve skeletal muscle function in this population. Likewise, 16 weeks of *n*-3 supplementation produced improvements in muscle function, although more modest in comparison to resistance training, but they caused notable improvements in markers of cardiovascular risk. Therefore, supplementation with *n*-3 PUFA rich in DHA might have the potential for decreasing cardiovascular risk and improving muscle function in postmenopausal women. Lastly, the modulation of myokine levels could be underlying some of the effects of DHA or RT; however, further research is necessary in order to clarify the regulation and role of each myokine.

## Figures and Tables

**Table 1:** Anthropometric characteristics of all postmenopausal women with overweight/obesity before the intervention.

Characteristic	Baseline
Age (years)	58.50 (3.20)
Weight (kg)	78.87 (7.35)
BMI (kg/m^2^)	30.61 (2.12)
Body fat (kg)	36.84 (4.83)
Body fat (%)	46.76 (3.29)
VAT (kg)	1.28 (0.46)
Lean body mass (kg)	39.59 (3.82)
Lean body mass (%)	50.42 (3.11)
Skeletal muscle mass (kg)	19.18 (2.54)
Muscle quality	10.73 (1.53)

BMI: body mass index; VAT: visceral adipose tissue; muscle quality calculated as 1-RM leg press + 1-RM chest press/skeletal muscle mass. *n* = 71, except for muscle quality = 61. Data are presented as mean (SD).

**Table 2:** Biochemical characteristics of all postmenopausal women with overweight/obesity before the intervention.

Characteristic	Baseline
Fasting glucose (mg/dL)	104.90 (15.50)
Insulin (mIU/mL)	9.96 (4.60)
HOMA-IR index	2.62 (1.44)
HOMA-β index	93.82 (47.65)
Triglycerides (mg/dL)	105.10 (43.60)
Total cholesterol (mg/dL)	245.60 (37.50)
HDL-cholesterol (mg/dL)	64.28 (14.70)
LDL-cholesterol (mg/dL)	160.36 (34.42)
VLDL-cholesterol (mg/dL)	21.03 (8.72)
Atherogenic index	−0.16 (0.23)
TNF-α (pg/mL)	1.02 (0.27)
IL-6 (pg/mL)	24.20 (9.67)
METRNL (pg/mL)	547.70 (162.10)
Myostatin (ng/mL)	9.23 (3.36)
Irisin (ng/mL)	12.68 (1.95)

HOMA-IR: homeostasis model assessment of insulin resistance; HOMA-β: homeostasis model assessment of β-cell function; HDL: high-density lipoprotein; LDL: low-density lipoprotein; VLDL: very-low-density lipoprotein; TNF-α: Tumor necrosis factor-alpha; IL-6: Interleukin-6; METRNL: meteorin-like; atherogenic index calculated as Log(TG/HDL-c). *n* = 71, except for the following variables: IL-6 = 49; METRNL = 65; myostatin = 70; irisin = 63. Data are presented as mean (SD).

**Table 3:** Effects of 16 weeks of DHA-rich *n*-3 PUFA (*n*-3) supplementation and/or resistance training (RT) on body composition, muscle quality, and glucose and lipid serum biomarkers in postmenopausal women with overweight/obesity.

Characteristic	P	*n*-3	P+RT	*n*-3+RT	*p* Two-Way ANOVA ^c^		
					*n*-3	RT	*n*-3xRT
**Body fat (kg)**							
Baseline	36.44 (4.24)	36.70 (5.09)	36.67 (5.58)	37.70 (4.62)			
Change	−2.92 (1.93) ^a,^***	−2.39 (2.02) ^a,^***	−2.39 (2.19) ^a,^***	−2.81 (3.14) ^a,^**	0.928	0.921	0.399
**Lean body mass (kg)**							
Baseline	38.16 (3.50)	41.28 (3.68)	38.93 (4.24)	40.64 (3.10)			
Change	0.29 (1.35)	−0.25 (0.77)	0.19 (0.93)	0.11 (1.11)	0.233	0.629	0.373
**Skeletal muscle mass (kg)**							
Baseline	18.21 (2.22)	20.31 (2.49)	18.78 (2.49)	19.83 (2.63)			
Change	−0.02 (1.25)	−0.29 (0.63)	0.12 (0.74)	0.17 (0.94)	0.618	0.182	0.485
**Muscle quality**							
Baseline	11.29 (1.47)	10.65 (1.89)	10.92 (1.56)	9.96 (1.11)			
Change	−0.05 (0.64)	0.40 (0.50) ^a,^*	1.18 (0.89) ^b,^***	1.77 (0.92) ^a,^***	**0.011**	**<0.001**	0.730
**HOMA-β index**							
Baseline	107.75 (44.19)	101.23 (66.96)	79.03 (39.60)	87.92 (36.18)			
Change	−13.28 (51.88)	−0.52 (43.52)	−5.56 (47.02)	−11.05 (30.89)	0.735	0.895	0.397
**VLDL-cholesterol (mg/dL)**							
Baseline	18.53 (5.89)	23.64 (11.06)	22.18 (10.33)	20.27 (6.65)			
Change	0.39 (5.26)	−5.77 (10.59) ^b,^*	−3.42 (4.76) ^b,^**	−3.78 (5.75) ^b,^*	**0.047**	0.576	0.075
Adjusted change ^d^	0.58 ± 1.10	−6.00 ± 1.74 ^b,^**	−3.53 ± 1.50 ^b,^**	−3.67 ± 1.67 ^b,^*	**0.039**	0.580	0.051
**Atherogenic index**							
Baseline	−0.22 (0.21)	−0.10 (0.23)	−0.14 (0.26)	−0.18 (0.19)			
Change	0.00 (0.11)	−0.11 (0.20) ^a,^*	−0.04 (0.09)	−0.07 (0.15)	**0.040**	0.917	0.204
Adjusted change ^d^	0.00 ± 0.03	−0.12 ± 0.04 ^a,^*	−0.04 ± 0.03	−0.07 ± 0.03	**0.037**	0.918	0.174

^a^ Paired Student’s *t*-test, ^b^ Wilcoxon’s signed-rank test. ^c^ Differences between groups for changes were evaluated by two-way ANOVA. ^d^ Means ± SEM adjusted by changes in body fat percentage. HOMA-β: homeostasis model assessment beta; VLDL: very-low-density lipoprotein; DHA: docosahexaenoic acid; PUFA: polyunsaturated fatty acids; Muscle quality calculated as 1-RM leg press + 1-RM chest press/skeletal muscle mass; atherogenic index calculated as Log (TG/HDL-c); P: placebo group; *n*-3: DHA-rich *n*-3 PUFA supplemented group; P+RT: placebo + resistance training group; *n*-3+RT: DHA-rich *n*-3 PUFA supplemented + resistance training group. Data are presented as mean (SD). *n* per group: P = 20; *n*-3 = 15; P+RT = 20; *n*-3+RT = 16; Muscle quality had 10 undetermined values; *n* per group: P = 16; *n*-3 = 12; P+RT = 18; *n*-3+RT = 15. The *p* values for the main factors of study, *n*-3 supplementation (*n*-3), exercise (RT), and the interaction between both (*n*-3xRT) appears under the corresponding column, with significant *p* values in bold. Statistical significance indicates a main effect for such factor (*n*-3: *n*-3-supplemented vs. P-supplemented groups; RT: RT vs. non-RT groups). * *p* < 0.05, ** *p* < 0.01, *** *p* < 0.001 vs. baseline.

## Data Availability

The original contributions presented in this study are included in the article/Supplementary Material. Further inquiries can be directed to the corresponding authors.

## Supplementary Materials

The following supporting information can be downloaded at https://www.mdpi.com/article/10.3390/nu17152553/s1, Table S1: Baseline characteristics of the postmenopausal women with overweight/obesity in the different experimental groups. Figure S1: Flowchart of the study. Figure S2: Protein intake before and after 16 weeks of intervention.
