# Supplementary material for: Myokine Circulating Levels in Postmenopausal Women with Overweight or Obesity: Effects of Resistance Training and/or DHA-Rich n-3 PUFA Supplementation
Source: Nutrients. 2025 Aug 5;17(15):2553. doi: 10.3390/nu17152553 (PMC12348652; doi:10.3390/nu17152553)
Supplement: Supplementary file 1 [file nutrients-17-02553-s001.zip › nutrients-3584951-supplementary.pdf]

SUPPLEMENTARY MATERIALS

Myokine Circulating Levels in Postmenopausal Women with Overweight or Obesity: Effects of Resistance Training and/or DHA-rich n-3 PUFA Supplementation

Alejandro Martínez-Gayo [1], Elisa Félix-Soriano [1], Javier Ibáñez-Santos [2], Marisol García-Unciti[1,3], Pedro González-Muniesa [1,3,4,*], María J. Moreno-Aliaga [1,3,4,*], on behalf of OBELEX Project.

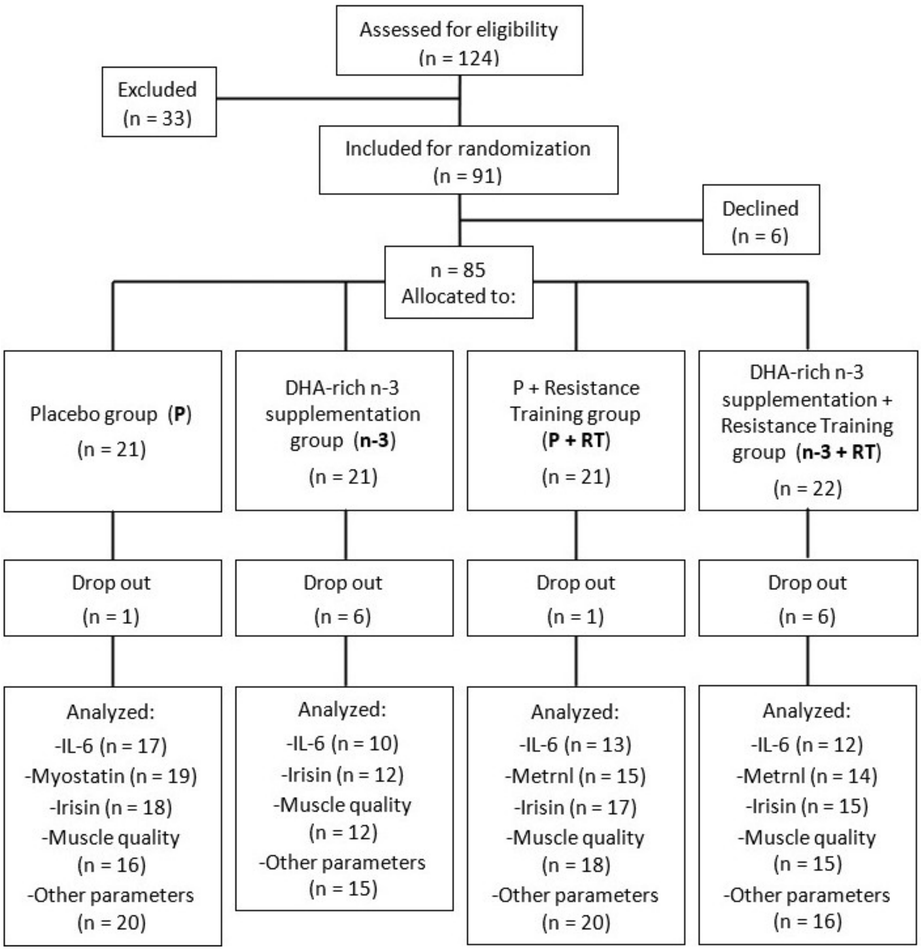

Figure S1. Flowchart of participants from the screening to the endpoint visit of the study, based on Félix-Soriano et al. (22). A total of 14 participants did not complete the study, due to health problems (n = 5, 3 unrelated to the study and 2 probably related to capsules consumption, one of them was related to gastroesophageal reflux and the other one related to itch in the hands), time incompatibilities (n = 2), withdrawal from the study (n = 3), and non-compliance with the training sessions (n = 4). Since data from some variables was not available for analysis (data not obtained or concentration of myokines not detected), the variables with a different n are specified.

Table S1. Baseline characteristics of the postmenopausal women with overweight/obesity in the different experimental groups.

Characteristic	P	n-3	P+RT	n-3+RT	*p* [a]
TNF-α (pg/mL)	1.04 (0.33)	1.00 (0.34)	1.06 (0.19)	0.94 (0.22)	0.588
IL-6 (pg/mL)	22.95 (5.60)	21.72 (3.55)	28.92 (16.29)	22.60 (5.86)	0.925
METRNL (pg/mL)	598.26 (205.06) [1]	528.61 (137.33)	604.10 (123.79) [2]	435.59 (86.72) [1, 2]	**0.003**
Myostatin (ng/mL)	9.22 (4.09)	9.11 (2.40)	9.71 (4.07)	8.76 (2.24)	0.925
Irisin (ng/mL)	12.10 (1.22)	12.94 (3.62)	12.97 (1.63)	12.83 (0.88)	0.139
Body fat (kg)	36.44 (4.24)	36.70 (5.09)	36.67 (5.58)	37.70 (4.62)	0.883
Lean body mass (kg)	38.16 (3.50)	41.28 (3.68)	38.93 (4.24)	40.64 (3.10)	0.099
Skeletal muscle mass (kg)	18.21 (2.22)	20.31 (2.49)	18.78 (2.49)	19.83 (2.63)	0.056
Muscle quality	11.29 (1.47)	10.65 (1.89)	10.92 (1.56)	9.96 (1.11)	0.075
HOMA-β index	107.75 (44.19)	101.23 (66.96)	79.03 (39.60)	87.92 (36.18)	0.123
VLDL cholesterol (mg/dL)	18.53 (5.89)	23.64 (11.06)	22.18 (10.33)	20.27 (6.65)	0.455
Atherogenic index	-0.22 (0.21)	-0.10 (0.23)	-0.14 (0.26)	-0.18 (0.19)	0.447

[a] Data analyzed by one-way ANOVA or Kruskal-Wallis depending on normality, followed by Tukey or Dunn's test. [1, 2] Means sharing the same superscript number were significantly different. P: placebo group; n-3: DHA-rich n-3 PUFA supplemented group; P+RT: placebo + resistance training group; n-3+RT: DHA rich n-3 PUFA supplemented + resistance training group; TNF-α: tumor necrosis factor-alpha; IL-6: interleukin-6; METRNL: meteorin-like; HOMA-β index: homeostasis model assessment of β-cell function; VLDL: very-low-density lipoprotein; Muscle quality calculated as 1-RM leg press + 1-RM chest press /skeletal muscle mass; Atherogenic index calculated as Log(TG/HDL-c). Statistically significant *p* values are marked in bold. N per group: P = 20; n-3 = 15; P+RT = 20; n-3+RT = 16; Number of undetermined values: IL-6 = 22; METRNL = 6; Myostatin = 1; Irisin = 8; Muscle quality: 10; n per group: P = 16-20; n-3 = 10-15; P+RT = 13-20; n-3+RT = 12-16. Data presented as mean (SD)

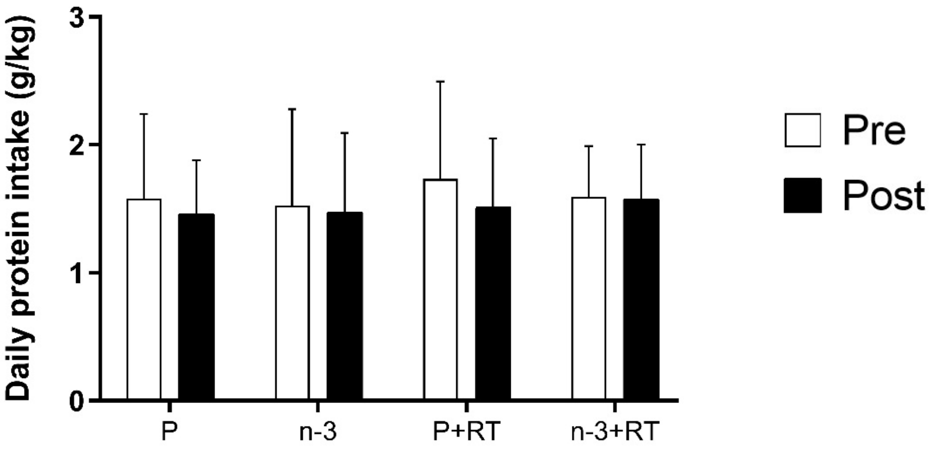

Figure S2. Protein intake in postmenopausal women with overweight /obesity, before and after 16 weeks of intervention with a DHA-rich n-3 PUFA supplement (n-3) and/or a RT program. P: placebo group; n-3: DHA-rich n-3 PUFA supplemented group; P+RT: placebo + resistance training group; n-3+RT: DHA-rich n-3 PUFA supplemented + resistance training group. N per group: P = 20; n-3 = 15; P+RT = 18; n-3+RT = 16. Comparison between protein intake levels before (Pre) and after the intervention (Post) analyzed by Paired Student's t-test or Wilcoxon's signed-rank test.
